# Guayusa (*Ilex guayusa* Loes.) Ancestral Plant of Ecuador: History, Traditional Uses, Chemistry, Biological Activity, and Potential Industrial Uses

**DOI:** 10.3390/molecules30132837

**Published:** 2025-07-02

**Authors:** Paco Noriega, Erick Moreno, Ana Falcón, Vanessa Quishpe, Patricia del Carmen Noriega

**Affiliations:** 1 Master Program of Natural Pharmaceutical Products, Universidad Politécnica Salesiana, Avenida 12 de Octubre N2422 y Wilson, Quito 170109, Ecuador; afalcons1@est.ups.edu.ec (A.F.); vquishpe@est.ups.edu.ec (V.Q.); 2 Group of Research and Development in Science Applied to Biological Resourses, Universidad Politécnica Salesiana, Universidad Politécnica Salesiana, Avenida 12 de Octubre N2422 y Wilson, Quito 170109, Ecuador; emorenop2@est.ups.edu.ec; 3 Ethnographic Museum, House of Ecuadorian Culture, Avenida 6 de Diciembre 345, Quito 170136, Ecuador; patricia.noriega@casadelacultura.gob.ec

**Keywords:** *Ilex guayusa*, stimulant, xanthine alkaloids, polyphenols, terpenes

## Abstract

One of the medicinal plants used in Ecuador that has the best prospects for industrialization is guayusa (*Ilex guayusa* Loes.). This review shows the potential of the species, analyzing the ethnobotanical aspects, ancestral uses, secondary metabolites, and research. The plant has been consumed for thousands of years by the high Amazonian peoples of Ecuador and currently forms part of the gardens of ancestral peoples and mestizo settlers. The most relevant secondary metabolites that have been investigated are xanthine alkaloids, terpenes, and phenolic compounds, while from the pharmacological point of view, the following uses stand out: physical and mental stimulants, analgesic, antioxidant, antimicrobial, anti-inflammatory, anti-diabetic, and phytohormonal. The goal of this review is to make known the benefits of guayusa, with the purpose of representing a resource that will provide benefits to the Amazonian inhabitants in the future.

## 1. Introduction

*Ilex guayusa* Loes. is commonly known as “Guayusa” in Ecuador and Colombia, with some variations in the Ecuadorian region such as “Guanyusa”, “Kirim”, “Waisa Waysa”, “Wais”, “Waís”, “Wayus”, “Wuayusa”, “Wayusa Panka”, “Aguayusa”, “Guafiusa”, or “Guañusa” [1,2,3,4,5,6,7]; in Peru, it is known as “Huayusa”, “Agracejo”, “Citrodora”, “Huitoc Quiro”, or “Vitoc Quiro” [1,4,8,9]. Guayusa is an evergreen tree [10,11], with a shrubby tendency in its juvenile stage [1], belonging to the Aquifoleaceae family [12]. A stimulating drink is prepared from its leaves [10,11,13], with medicinal active ingredients, among which caffeine is the most important [14,15]. In addition, there are other molecules with bioactivity such as polyphenols and terpenes [16,17,18].

Its specimens are mainly distributed in the Amazon region of Ecuador [17], with a presence in neighboring sectors in Colombia and Peru [2,11,19], while collections exist in Venezuela and Bolivia, which are probably of imported species [20,21,22]. In Ecuador, the species is domesticated and cultivated in home gardens [2].

**Figure 1:**
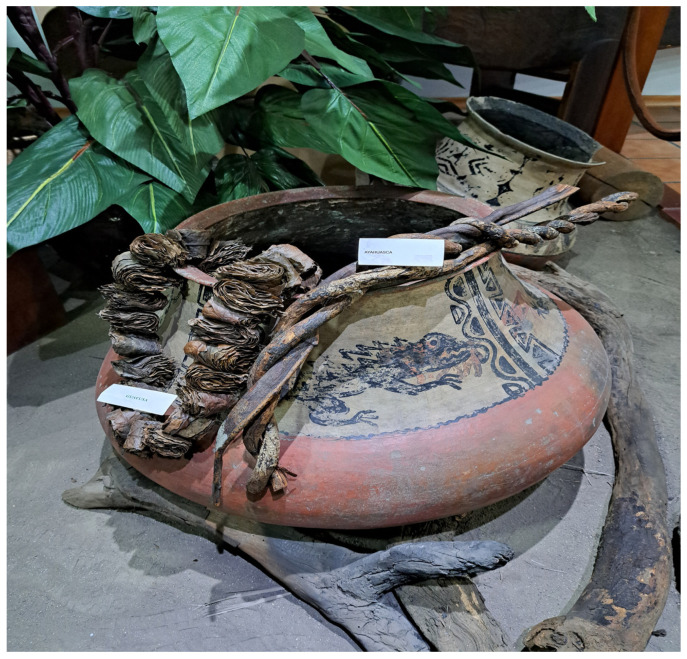
Traditional form of presentation of guayusa, forming a necklace of leaves.

The most widely used plant segment, due to the concentration of secondary metabolites and their bioavailability, are the leaves [2,22,23], which are traditionally marketed in packages tied with string in the form of a necklace, after having been dried at ambient temperatures—Figure 1.

Similar to other species of the genus Ilex, its aqueous or alcoholic infusions are very appetizing [2,11,24]. The preparations have a pleasant taste with a sweet touch and an invigorating effect [17,25], similar to that provided by another very popular drink in South America, “hierba mate”, Ilex paraguariensis [26]. While it is true that the most widespread use of guayusa infusions is as a stimulant, the plant has other uses in medications and treatments for hypoglycemia [4,9], monthly colic and fertility [27,28,29,30], respiratory diseases [2], reducing fever [7], joint pains [7], malaria [7], gastritis [28,31], diarrhea [29,30,32], stomach pain [27,30,32], venereal diseases [2,27,30], prostate inflammation [27,30], appetite suppression [7], purgatives [27,30], diuretics [2], and brain tonics [7]. As a ritual plant it is used in detoxifying steam baths [2], and as an additive to the entheogenic drink known as “Ayahuasca” [7,12,22].

The bioactive molecules described in guayusa are the following: alkaloids, carotenes, triterpenes, coumarins, tannins, flavonoids, and simple phenols [1,2,10,12,26,30]. As it is a plant that has been domesticated for millennia, its use for local consumption, as well as industrially, does not compromise its survival [20].

This review seeks to investigate the pharmaceutical, cosmetic, and food potential of guayusa, which is essential for proposing the species as a resource that brings development to the communities of the Amazon region of Ecuador, with sustainable exploitation.

## 2. Results

### 2.1. Market Perspective and Economic Potential

Due to the interesting biological activity and active principles of guayusa, industries such as cosmetics, pharmaceuticals, nutraceuticals, and food could benefit from its properties [15,18,23,31,32]. Additionally, the sale of raw material or the elaboration of stable extracts for direct commercialization could be profitable [33,34]. Currently, most of the cultivated plant is used in the production of energy drinks, infusions, snacks, and cereal bars, among others [27,32,35].

The guayusa market in 2024 was valued at USD 9.63 million, with projections to reach USD 12.05 million by 2029, with an annual growth rate of 4.6% for the period 2024–2029 [36]. An important challenge facing the industrialization of guayusa is the lack of knowledge of its properties outside its region of origin [32]. Ecuador stands out as the world’s largest producer, covering about 98% of the market. In the provinces of Napo, Pastaza, and Orellana, in 2022, production values of 72 tons per year were determined, under the model of 625 plants per hectare, generating more than 3 million USD in guayusa leaf exports, which boosted the development of indigenous communities through agroforestry systems, complying with organic production and fair trade standards and promoting the preservation of the environment [33,35]. Official records report the beginning of guayusa exports from Ecuador in 2010, mainly to the United States, as well as, by 2015, Japan, Germany, United Kingdom, and Italy. This list has increased progressively, having, by 2024, 25 countries registered as buyers [33]. There are several companies that distribute guayusa or its derivatives, mostly in the form of energy drinks or infusions [32]. In Ecuador, this includes Ami Runa, Paccha, Univfood, Cetca, Tryskelwork, and Waykana Tea Company/Danec [35], who formed the first consortium of guayusa exporters in the country in 2022 [33]. Other key companies that have entered this market are: Yamotoyama Co., Ltd., Doehler (Teawolf), Stash Tea Company, Garden Flavor, Herbs America Inc, and Bi nutraceuticals [32,36].

Guayusa is grown in small vegetable gardens, known as “Chakras”, which are environmentally friendly and consistent with the ancestral practices of the Amazonian communities of Ecuador [28]. An example of this is the Wiñak Artisanal Farming Association, from the community of the same name, which sells dried and crushed leaves on demand in small and large quantities [37].

### 2.2. Botany

Guayusa is a dioecious species, which, despite being able to form seeds in the areas where it is cultivated, reproduces mainly by hardwood cuttings or cuttings from selected mother plants. The fertility of the seeds could be affected by the effect of domestication through vegetative propagation, embryonic immaturity, or the absence of specific pollinators due to changes in its habitat [1,2].

Native to the tropical rainforest, it is classified as an Andean Amazonian species, developing at altitudes ranging from 0 to 1500 m.a.s.l. [9,19], with exceptional reports up to 2600 m.a.s.l. [1,21]. Guayusa plants are mainly found in the Ecuadorian Amazon [27].

**Figure 2:**
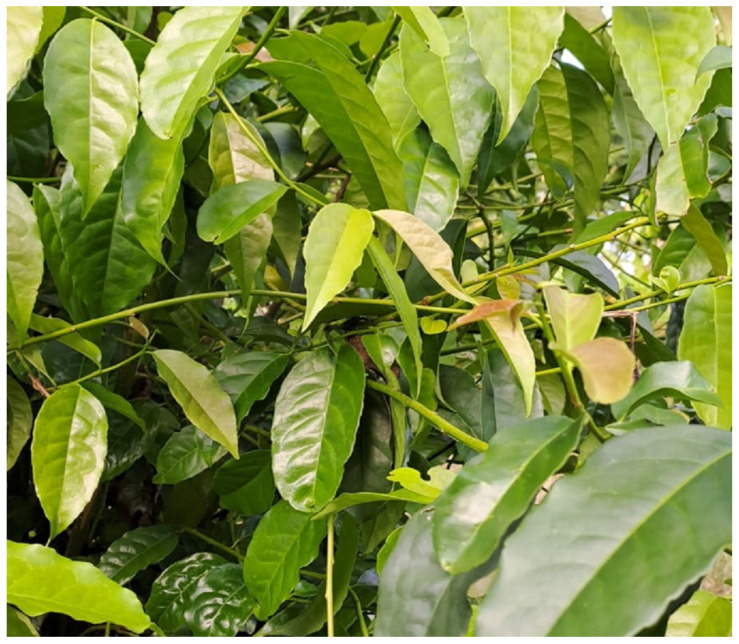
*Ilex guayusa* young plant from Ecuador’s Amazonian region.

Its leaves have an alternate arrangement, with a sharp apex and a sharp base connected by a 1 cm petiole. They are simple, toothed, oblong–elliptical in shape, leathery, olive green, and glabrous or subglabrous on both sides. They can grow to 15–21 cm long and 5–7.5 cm wide [1,10]. Its flowers have a persistent calyx, a white-greenish corolla, obtuse petals that are found in the same number as the stamens, oblong anthers, a sessile ovary, and a subglobose segmented with 4–6 loculi. The fruit produced is a globular green berry 1 cm wide [1,10]. Domesticated individuals report an average of 10–15 m in height [1,2], while the main trunks can reach a diameter at chest height of up to 50 cm [2,23]. In wild trees, heights of up to 25 m have been detected and the width of their trunks at chest height of up to 80 cm [2,23]. *Ilex guayusa* has irregularly shaped leaves and characteristic dense foliage, while the trunk is usually forked at chest height and has a smooth texture and white bark on the trunk and branches, which are spread out and quite flexible [10]. Figure 2 shows a young guayusa tree in its habitat.

Despite being categorized as a durable heliophyte, it can survive in low light levels, with semi-dark areas even being recommended, as they stimulate the development of seedlings and the proliferation of branches [1]. The compensatory strategy of increasing ramifications in low light conditions is due to the fact that basal shoots receive less radiation, hence they die or lose vitality, as do other plant species under the canopy, which provides a bed of leaf litter and logs that, after decomposition, provide essential nutrients for the development of the plant [1].

### 2.3. Ecology

It develops under conditions typical of the Amazonian ecosystem: in eroded acidic soils with a pH between 4.3 and 5, low nutrient availability, low cation exchange capacity, sandy loam texture, high moisture retention, high aluminum content, the presence of heavy metals [1,38], and solar spectrum abundant in green and far-red light [28]. The conditions also comprise a high humidity, average temperatures of 24 °C, and persistent rainfall of 2000 to 5000 mm per year [38].

In the wild, it has a marked association with the species *Tabebuia insignis* and *Mauritia flexuosa* with which it establishes a vegetation unit that forms a small- or medium-sized forest, with a short basal area, high shrub density, and a considerable number of palms that stand out from the grouping [1].

### 2.4. In Vitro Reproduction

In order to maintain the permanence of this species over time, in addition to responding to the growing demand for medicinal plants globally, effective and sustainable proposals for in vitro propagation have been developed [39]. Considering that the efficiency of vegetative propagation is only 50% and the expansion of cultivation areas may pose a threat to the tropical forest, laboratory propagation represents a good long-term alternative [28].

The viability of in vitro propagation has been evaluated using stem cuttings as explants, with sterilization with ethanol and sodium hypochlorite; mWPM medium (modified Woody Plant Medium) for shoot development, elongation, and rooting; and activated carbon to reduce browning, with good results. To stimulate this process it is recommended to use indole-3-butyric acid auxin or indole-butyric acid (IBA), in addition to considering the photobiology of the species, where green or far-red spectra promote spontaneous root development [28].

### 2.5. Ancestral Uses of Guayusa

#### 2.5.1. History

The first reports of the handling of this plant date back to the year 500 of the contemporary era, but it is speculated that its cultural use dates back much earlier thanks to the archeological findings found in the province of Bautista Saavedra in Bolivia inside a multifamily tomb, where bags with carefully prepared guayusa leaves were found along with other instruments such as enemas, trays and snuff tubes, a human skull, spatulas, and mortars, among others, presumably belonging to a healer of the “Kallawaya” or “Tiwanaku” ethnic group [2,40]. The guayusa leaves found give indications of its high value at the time, since the species does not report a presence near the area where they were found, so it is intuited that it was used as a tradable medicine or as an energy source for long trips due to the stimulating effect of the alkaloids it contains [2,27,41].

During the Spanish colonial period, there are records from the eighteenth century be-longing to religious missions, which detail the applications of this plant in communities, such as the Santa Rosa mission in Colombia, where multiple medicinal uses are de-scribed, such as treatment of venereal diseases, in addition to the consumption of the infusion mixed with honey to treat infertility. Jesuits in Ecuador, who, in addition to keeping records of the usefulness of guayusa, marketed and popularized its leaves in Quito as a cure for sexually transmitted diseases [2,40].

The first description and botanical classification of guayusa was made by Theodor Loesner, but it was without floral material, so it was quite controversial and considered incomplete [31]. In 1857, Richard Spruce, a British explorer, conducted intensive research on guayusa reporting the first records of plantations of this species in the historic area of Antombós, near the city of Baños-Ecuador, as well as detailing purging rituals before the start of the day for the first time [2].

Ecuador is the country with the largest number of records of ritual practices and guayusa consumption [5,27,42]. Amazonian ethnic groups use it for its stimulating properties, attributing to it various health benefits. It is also used for the treatment of supernatural diseases and common ailments associated with the presence of evil spirits [27].

#### 2.5.2. Uses of Guayusa by the Amazonian Kichwa People

For these people, guayusa is considered a sacred plant which prior to its vegetable form was a divine being with qualities associated with the effect produced by the consumption of its infusion, such as spiritual and physical strength, assertiveness for decision making, vitality, robustness. They also considered the plant as a reference of fertility, libido, and victory [27,43].

The consumption of guayusa is carried out as a tradition of coexistence. Early in the morning, the preparation of the infusion is entrusted to the daughters-in-law of each family group who serve the drink in natural containers. While drinking the decoction, participants tell stories, interpret dreams, dance, pray, weave, and perform rituals to reorient the younger members. In addition to the usual consumption, events are held taking this plant as the central axis, such as the “Great Guayusazo Bailable” (Dancing Guayusazo) [2,7].

In combination with ginger, lime juice, “chuchuwasi”, and sugar cane liquor, the Kichwas use the guayusa infusion to treat colds, or without combining it, as a stomach tonic, to soothe body aches or clean their mouths. They also use this decoction on the skin as an insect repellent, to prevent or treat snake bites, and delay aging [2]. This relationship with guayusa is not limited to the Kichwa people living in the Amazon; Andean Kichwas of the Ecuadorian highlands adopted the use of guayusa as a medicinal plant to treat various ailments, focusing on pain relief [18], and also incorporated in the preparation of alcoholic macerates like their lowland relatives [2,7].

#### 2.5.3. Uses of Guayusa by the Achuar People

These people use guayusa in the ritual known as “Wayus”, the objective of which is to strengthen community ties, purify the body, and communicate with the material and spiritual worlds. The ceremony consists of ingesting copious amounts of guayusa decoction before dawn and then inducing vomiting [2,10,14,27]. The Achuar consider it improper for a man to start the day with a full stomach so they induce vomiting, as well as avoid the adverse effects produced by excessive caffeine consumption, such as headaches, nervousness, tremors, and distorted perceptions that interfere with hunting and threaten their safety [14]. For women, the volumes consumed are lower and the emesis is not a custom [14]. The two liters consumed by each individual in the ceremony are obtained from a decoction prepared under the approximate ratio of 150 g dry leaves in 20 L of water, presenting a concentration of methylxanthines of approximately 2.64%, mainly constituted by caffeine, which would be the equivalent to consuming 5.5 cups of coffee [14].

The leaves used for the different rituals come from selected specimens, since not all have the same concentration of secondary metabolites. Due to genetic and physiological variations being discarded, the “wild” specimens that have the highest concentration of methylxanthines are not usually propagated [14].

#### 2.5.4. Other Ancestral Peoples

For other peoples, the consumption of guayusa is also part of their identity, being used mainly as a stimulant and for pain relief, as in the “Shuar” ethnic group, who drink the infusion for its multiple benefits but do not incorporate it as a daily ritual activity, the “Secoya” people who use it to treat physical ailments, the “Cofán” ethnic group who use it extensively in the preparation of various drinks, and the “Tsa’chi” community, where it is drunk and the leaves are used in steam baths, for medicinal purposes, to relieve menstrual issues [2,7].

In the Shuar, Awajun, and Achuar peoples, its ritual use is emphasized in ceremonies such as the Women’s Tobacco Ceremony, the Festival of Victory, or the Feast of the Tsantsa, where it is mainly used for spiritual cleansing [2,40]. In mestizo settlements in eastern Ecuador, the infusion is consumed for pleasure, usually mixed with lemon juice and sugar. The leaves are also used as an additive in the preparation of liqueurs [2,10]. In the city of Macas, they serve a drink called “guayusa con hueso”, prepared by mixing a decoction of guayusa with an alcoholic macerate called “Chuchuhuazo”, obtained from the species *Maytenus krukovii* [10].

#### 2.5.5. Other Uses

Another rather particular use of the guayusa infusion is related to hunting dogs, which, while for the Shuar groups are a gift from “Nukui” (mother earth), for the Kichwua they are a gift from “Sachahuarmi” or “Sacharuna” (spirits of the forest). Both ethnic groups wash the heads of their animals or administer small doses to them nasally to im-prove their hunting skills, acting as a stimulant and psychoactive, sharpening their state of alertness, and helping them to dream so that by interpreting these dreams they can forecast the hunt [2,6,44,45].

### 2.6. Guayusa Secondary Metabolites

The main difference between guayusa and other members of the genus Ilex, such as *Ilex paraguariensis* (yerba mate) and *Ilex vomitoria* (yaupon), is its high xanthine alkaloids content [27], mainly caffeine, with the highest value reported for plant leaf tissue (75.7 mg of caffeine/g dry matter) [11,14], which is only equaled by guarana seeds (*Paullinia cupana* Kunth var. *sorbilis*) with 78 mg of caffeine/g dry matter [11,46]. It should be taken into account that for the Ilex genus there can be very marked metabolomic chemical diversity between members of the same species due to the influence of the environment, location, climate, soil, specific conditions, age, harvesting method, genotypic variation, and genetic intro-gression [11,47].

Guayusa also stands out due to the presence of phenylpropanoids, chlorogenic acid, caffeic acid, and their derivatives [31]. Qualitative phytochemical analyses, known as “phytochemical screening”, show the presence of alkaloids, sesquiterpene lactones, coumarins, cardiotonic glycosides, steroids, terpenes, carotenoids, flavonoids, tannins, and saponins [22,27]. Several guayusa metabolites have been quantitatively analyzed using spectrophotometric techniques, high performance liquid chromatography (HPLC), and liquid or gas chromatography coupled with mass spectrometry. Among the molecules evaluated, the following stand out: polyphenols, flavonoids, and alkaloids [11,13,15,17,31,34,48].

The bleaching process, which consists of preheating the raw material by immersion in water or steam followed by rapid cooling in order to inactivate enzymes, was shown to be effective for the preservation of polyphenols and carotenoids. Furthermore, as the increase in temperature and humidity, followed by cooling, can cause cell rupture, it is also possible to increase the release of carotenoids and phenols [18,49]. Using modern extraction techniques, such as supercritical fluid extraction, it has been possible to isolate and analyze terpenes such as squalene and α-amyrin [31].

#### 2.6.1. Alkaloids

**Figure 3:**
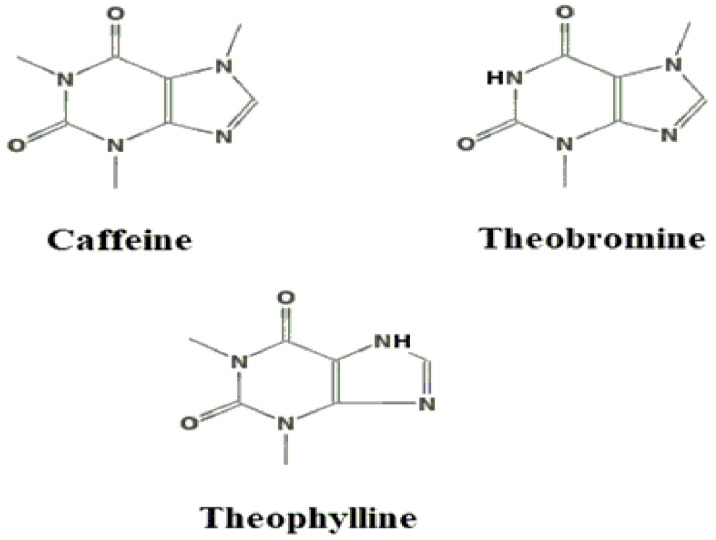
Xanthine alkaloids from *Ilex guayusa*.

These are the metabolites with the best-known effects in guayusa, including physical and mental stimulation, diuresis, and increased heart rate [13]. Three xanthine alkaloids have been detected in guayusa: caffeine, theobromine, and, in small quantities, theophylline—Figure 3 shows their structures. These have been analyzed using various instrumental methods of chemical analysis [10,11,13,31,34].

Caffeine concentrations vary depending on the extraction method. For decoctions and infusions, they exceed 1%, reaching 3% [13,14]; using ultrasound the value is between 1.92% and 2.69% [11]; 1.22% using Soxhlet extraction [31]; and 0.85% by ethanol extraction [13].

Theobromine has a lower concentration, ranging from 0.02 to 0.12% in decoctions [14] and measured as 0.008–0.025% according to ultrasound [11]. In general, theophylline is found in very low concentrations, between 0.002 and 0.005 mg/g, using ultrasound extraction [11]. Following the ancestral preparation used by the Amazonian Achuar groups, after boiling the guayusa leaves for 1 h, a concentration of around 3% caffeine is obtained, but when using leaves from wild specimens the concentration of this alkaloid can reach up to 7.57% [11,14].

#### 2.6.2. Terpenes

Among the most abundant compounds in guayusa, after the alkaloids, are pentacyclic terpenoids, mostly consisting of ursolic acid with a range of 0.7–1% and amyrin ester complexes representing more than 0.5% [15]; α-amyrin palmitate is the main constituent of this second group with 0.02–0.39 mg/g in extracts obtained by supercritical fluids or Soxhlet extraction [33]. Another representative terpene is squalene, with a concentration between 0.09 and 0.95 mg/g [33].

**Figure 4:**
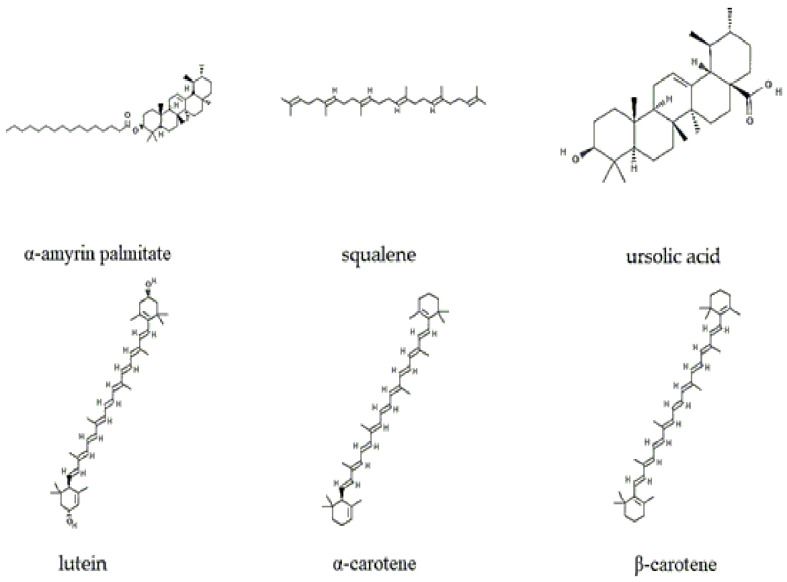
Most abundant terpenes of *I. guayusa*.

Another group of terpenes found in guayusa is carotenoids. Using the high-performance liquid chromatography technique, the following carotenoids were detected and quantified: lutein with a concentration of 173.38–266.33 μg/g; β-carotene with 37.85–115.59 μg/g; α-carotene with 46.76–113.12 μg/g; violaxanthin 8.17–108.46 μg/g; and neoxanthin 8.17–108.46 μg/g—Figure 4 shows the most abundant terpenes. The concentrations vary depending on the treatment of the guayusa, whether it comes from green leaves or fermented leaves [18].

#### 2.6.3. Phenolic Compounds

Guayusa is characterized by a high concentration of polyphenols, especially flavonoids [13,50], the most abundant of which are chlorogenic acid, quercetin, and kaempferol [18,23,51]. Other polyphenols reported are 3-hydroxyflavone and tectochrysin, which were detected by gas chromatography coupled with mass spectrometry and previously derivatized with a silylation reaction [23]. Phenols are obtained by decoction of the leaves [17,52], with excellent results at a temperature of 70 °C and a time of 8 h [17,31]. The use of ultrasound also increases the concentration of polyphenols [18].

There are several studies that evaluate polyphenols and flavonoids as total groups [18,23,31]. The most commonly used methods are: Folin–Ciocalteu, with gallic acid standard curves (total phenols) [18,23,31]; aluminum chloride, with quercetin curves (total flavonoids) [27]; and the Lamaison–Carnat method, with a hyperoside standard (total flavonoids) [23].

The types of extracts studied have been acoustic, alcoholic, or mixed [13,18,23,51,53] and the extraction methods diverse, such as decoctions, macerations, Soxhlet extractions, and supercritical fluids [31].

As a result of the quantification of total phenols, the following values have been measured: 57.7 mg gallic acid/g in total ethanolic extract [27]; 54.86–106.62 mg gallic acid/g depending on the treatment of the leaves [18]; 54.39–67.23 mg gallic acid/g depending on the fermentation process [13]; 54 mg gallic acid/g using ethanol and 33 mg gallic acid/g using ethyl acetate [53]; 33.44 mg gallic acid/g for 2-month-old leaves and 21.42 mg gallic acid/g for 6-month-old leaves, by extraction with 70% acetone [51]; 4.9–33.3 mg gallic acid/g depending on the conditions and solvent used [54]; 20.18–22.4 mg gallic acid/g depending on the solvent [23]; 22.33 mg gallic acid/g in ethanolic extract by Soxhlet extraction and 0.28–4.04 mg gallic acid/g using supercritical CO_2_ extraction, depending on the conditions and solvents [31]; and 0.033–0.055 mg gallic acid/g in an aqueous extract [34].

Regarding the amount of total flavonoids, the following values have been obtained: 3.8–7.5 mg hyperoside equivalents/mL depending on the percentage of ethanol; 7.40 mg hyperoside equivalents/mL in hydroglyceric extract, using the Lamaison–Carnat method [23]; or 1.71 mg quercetin equivalents/g in total ethanolic extract, using the aluminum chloride method [27]. The high variability found in the concentration values, which has been previously reported, can be attributed to the solvent, analysis technique, extraction method, particular characteristics of the material used, or particularities of the plant from which the leaves were obtained [11,31,34].

Among the flavonoids, the compounds of interest reported in the aqueous extract with their concentration values are: catechin 2 mg/g, epicatechin 0.179 mg/g, epicatechin gallate 0.199 mg/g, epigallocatechin 1.11 mg/g, and epigallocatechin gallate 0.0876 mg/g [55].

Other phenolic compounds that are not flavonoids of bioactive interest are: chlorogenic acid with values of 7.53–26.53 mg/g in hydroalcoholic extract [18], 6.42–6.55 mg/g in aqueous extract, and 4.48–5.14 mg/g in alcoholic extract [17], reporting higher concentration than coffee [17]; together with neochlorogenic acid with measurements of 2.55–11.30 mg/g in hydroalcoholic extract [18], 3.46–3.58 mg/g in aqueous extract, or 3.06–3.31 mg/g in alcoholic extract [17], and its derivatives (esters of hydroxycinnamic acids) [13,18] having the concentration values of the most abundant compound of each group reported [17].

**Figure 5:**
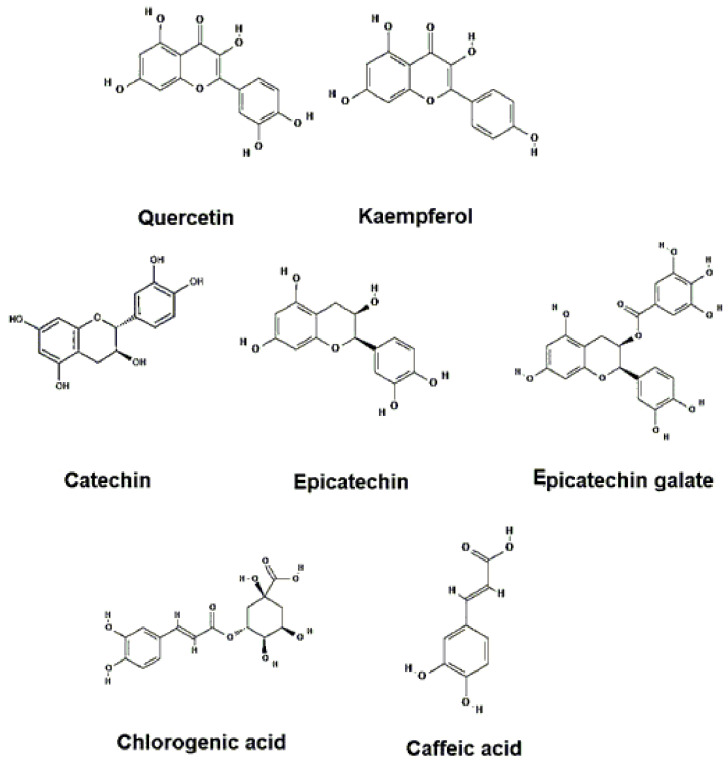
Most abundant Phenols and Flavonoids in *I. guayusa*.

Among the caffeic acids and derivatives are the following: caffeic acid glycoside-1, with 0.11–0.12 mg/g in water and 0.11–0.14 mg/g in alcohol [17]; coumaroylquinic acids [13], with a higher concentration in alcoholic extracts; 2-p-coumaroylquinic acid, with 0.099 mg/g water and 0.12 mg/g in alcohol [17]; feruloylquinic acids [13], with higher solubility in alcoholic extracts; 3 feruloylquinic acid and 5 feruloylquinic acid, with values of 0.07–0.11 mg/g in water, 0.08–0.12 mg/g in alcohol, and 0.07–0.13 mg/g in water, 0.10–0.16 mg/g in alcohol, respectively [17]. Figure 5 shows the phenolic compounds and flavonoids with the highest presence in guayusa.

All of the above phenolic compounds are of high interest for their bioactivity and are potential natural products for various industries, such as pharmaceuticals, cosmetics, and functional food [56]. Figure 4 details the phenolic and polyphenolic compounds with high concentrations in the guayusa plant.

### 2.7. Guayusa Biological Activity

Most of the health-beneficial effects provided by the consumption of this species are determined by its flavonoid and polyphenol content [13,18,51], while the stimulant effect is mostly due to its high concentration of alkaloids, mostly composed of caffeine [10,11,14]. The efficiency of biological activity is directly linked to the extraction method used, the solvents applied [31], the processing techniques such as bleaching or fermentation, and the conditions and age of the raw material [18,51], as well as the metabolomics of the specimen from which the leaves were obtained [11]. Another relevant aspect to consider, apart from the phytochemical composition, is the bioavailability of the compounds upon ingestion of the infusion, since the efficiency and magnitude of the physiological effects is derived from this [17].

Several parameters can have a direct impact on the bioactivity of guayusa extracts, such as antioxidant activity, where pH variations increase the efficiency of this property. Equally important is the solvent used in the extraction, which, depending on whether it is alcoholic or aqueous, allows certain interactions capable of stimulating the degradation or formation of molecules, altering the bioavailable quantities, mainly by enzymatic action [17,57,58,59].

Guayusa is a safe plant for consumption; evaluating its infusions presents toxicity levels with lethal concentration values of (LC_50_) > 10,000 µg/mL in aqueous extract and 300 µg/mL in alcoholic extract, determined by the lethality test in *Artemia salina* (BSLA) [60], while the mean lethal dose (LD_50_) presents a value >5000 mg/kg body weight, determined in female albino rats [55]. Safety evaluations have been carried out, such as the bacterial reverse mutation test (Ames test), which determines the mutagenic potential, or the chromosomal aberration test in human peripheral lymphocytes, which determines the clastogenic capacity; these evaluations have been reported to be negative for the aqueous extract of guayusa leaves [55].

#### 2.7.1. Physical and Intellectual Stimulant

The stimulant effects produced by the consumption of guayusa are directly linked to the most abundant secondary metabolites, the xanthine alkaloids, which, as mentioned, are mainly composed of caffeine (1, 3, 7 trimethylxanthine), a molecule that has a direct effect on the central nervous, cardiovascular, and pulmonary systems [10,55]. The stimulation it causes in the nervous system consists of vasodilatory effects on peripheral blood flow, vasoconstrictor effects on cerebral circulation, and antagonistic effects on the level of adenosine receptors [55]. Due to its lipophilic profile that allows it to cross the blood–brain barrier, it has the ability to reduce fatigue, increase alertness, and accelerate the heart rate. It has a very particular pharmacokinetic–pharmacodynamic effect, which becomes dependent on the genetics of the individual who consumes it [10,61,62].

Caffeine, together with other methylxanthines, mainly theobromine, are highly studied molecules, which enhance their action in a synergistic manner by preserving their natural composition, reportedly retarding the aging of cognitive function, improving mood and alertness, and stimulating the efficiency of brain skills, such as concentration, memory, or learning [29]. An interesting bioactive compound present in guayusa and involved in brain function is theobromine, the second most abundant methylxanthine, which causes effects on the peripheral nervous system such as broncho-vasodilation, decreasing blood pressure, and increasing heart rate [62]. This alkaloid is able to reduce reaction time [63], improve motor learning [64], and increase alertness, energy and motivation, although in some cases it can cause drowsiness [29].

Another group that stands out in this area, besides alkaloids, are phenols, specifically chlorogenic acid, the most abundant compound of this group, a procognitive agent of high relevance and anxiolytic properties [65] that is able to protect neurons [66], improve endothelial function through the release of nitric oxide and thromboxane A2 [66], recover memory after cognitive impairment caused by diabetes, scopolamine, or ischemia [66], in addition to enhancing learning, spatial memory, and motor function [29].

Within the phenols, we have the subgroup of flavonoids, which have a high antioxidant capacity [29], produce peripheral effects similar to those generated by alkaloids, such as vasodilation and by increasing cerebrovascular blood flow, and improve oxygenation with procognitive effects [29,67]. Catechins categorized within flavonoids are very relevant compounds, such as epigallocatechin 3-gallate which has an outstanding antioxidant action and is able to improve endothelial function and increase nitric oxide supply [68].

#### 2.7.2. Antioxidant and Anti-Aging Activity

Antioxidant molecules decrease the probability of suffering from diseases associated with oxidative stress [17], such as atherosclerosis, Alzheimer’s disease, cancer, or chronic obstructive pulmonary syndrome, to mention a few [69].

The main antioxidant phenolic molecules are mono- or di-substituted caffeoylquinic acids, together with their derivatives [13,18]. Also chlorogenic acids have had numerous reports of their antioxidant, anti-inflammatory, anti-obesity, chemopreventive, and cardiometabolic-reducing capacity, or of their role as an adjuvant in the treatment of type 2 diabetes and Alzheimer’s disease [18].

Other antioxidant molecules are carotenoids, with health benefits such as improved immune response, a reduced risk of degenerative diseases, anticarcinogenic action, and the treatment and prevention of cardiovascular diseases, macular degeneration, and cataracts; lutein, the most abundant carotenoid in guayusa, is particularly effective in reducing damage associated with vision [18].

For the particular case of guayusa, there are reports of a positive correlation between polyphenol–carotenoid content and antioxidant capacity [18,31], as well as for other species that express a positive relationship between TPC and DPPH parameters, so further studies are required to determine whether the high antioxidant capacity is due to the concentration of phenols, carotenoids, specific compounds, or another particularity [17,70,71].

In the case of guayusa leaves, the antioxidant capacity, which is already quite high, is enhanced by the increase in infusion time and temperature, regardless of the solvent used, and it is advisable to perform the decoction procedure [13,31]. The increased solubility of the bioactive components of the infusion may contribute significantly to the antioxidant capacity, and to the content of total phenols [17].

#### 2.7.3. Analgesic and Anti-Inflammatory Properties

Inflammatory processes can act as an aggravating factor or have a negative impact on other conditions, and can even cause insulin resistance, leading to heart problems [72].

There are several systems where the compounds present in guayusa can act to enhance the anti-inflammatory action; one of them is the endocannabinoid system, where 2-arachidonoylglycerol (2-AG), which is used to treat chronic and inflammatory pain, enhances its action thanks to terpenic compounds such as amirins, which have a high concentration in guayusa [15,73]. Amirins inhibit the enzymatic degradation of this endocannabinoid, thus contributing to its analgesic and anti-inflammatory role [73].

Another anti-inflammatory function of guayusa’s secondary metabolites is the elimination of nitric oxide (NO) and the suppression of its cellular production. Nitric oxide is a naturally produced reactive free radical that plays an important role in cell signaling and pressure regulation, but it can be produced in abundance, causing persistent infections, excessive inflammation, as well as the formation of highly destructive compounds such as peroxynitrite [13].

The inhibitory action of NO production, as well as the antioxidant action, is associated with caffeoylquinic acids, such as chlorogenic acid and 3,4-dicaffeoylquinic acid [13]. Guayusa beverages report a NO removal efficiency of 18.16–24.38%; this property increases proportionally with the infusion time [13]. For the suppression of NO production, evaluated in the RAW 264.7 cell line from mouse macrophages, guayusa extracts report a capacity of 10.30–27.21%, being more efficient in this assay than the non-fermented extracts obtained with prolonged infusion times [13,18].

#### 2.7.4. Metabolic and Immunostimulating Effects

The action exerted by guayusa on the metabolism is largely due to caffeine and ursolic acid, while methylxanthine is able to increase the metabolic rate or promote fat oxidation by various mechanisms associated with its stimulant action [29,55,61], while pentacyclic triterpene plays a key role in combating diabetes, obesity, and associated pathologies [15,74,75].

Human studies show that caffeine consumption, in addition to increasing metabolic rate, promotes fat oxidation through direct lipolysis and catecholamine release [55]. Caffeine and catechins have a synergistic anti-obesity effect, while theophylline can change blood composition, increasing cholesterol, glucose, and uric acid [55].

The anti-diabetic effects of this plant are linked to its antioxidant and anti-inflammatory capacity, since oxidative stress, inflammatory phenomena, and vascular abnormalities are involved in the pathogenesis of this disease [13,76]. The components with anti-diabetic action are ursolic acid and amyrin–ester complexes [15].

Ursolic acid acts by activating the membrane receptor TGR5, which is linked to the management and prevention of several metabolic syndromes such as diabetes, obesity or fatty liver [75,77], as it regulates insulin sensitivity, in addition to several processes related to lipid metabolism, glucose homeostasis, and energy expenditure [15,75]. Upon activation of the membrane receptor by ursolic acid, which is more efficient than the conventional activator betulinic acid, incretin secretion increases, which stimulates insulin production. Incretin in the form of glucagon-like peptide type 1 (GLP-1) reduces blood glucose levels [78].

The amyrin mixture also exhibits anti-diabetic action by acting as selective enzymatic inhibitors of the degradation of the endocannabinoid 2-arachidonoyl glycerol (2-AG), contributing to glucose uptake and insulin sensitivity [72,73].

Another compound present in guayusa used for the treatment of type II diabetes mellitus is guanidine, whose biguanide-type derivative (called galagenin), like metformin, is commonly used to treat this condition, which acts as a hypoglycemic drug, reducing blood glucose [22,79,80]. Guanidine derivatives not only have functions in metabolic regulation through various molecular interaction mechanisms, but thanks to their versatility from being highly susceptible to structural modifications, they possess a broad spectrum of biological functions, acting as antitumor, antifungal, antiprotozoal, and antiviral agents [81,82].

#### 2.7.5. Antimicrobial Activity

Evaluations of antimicrobial activity indicate a high inhibitory potential against several pathogenic microorganisms such as *Staphylococus aureus*, *Bacillus subtilis*, and *Klebsiella pneumoniae*, with better results using infusions prepared in water compared with ethanolic extracts, and presenting greater effectiveness in combating Gram-positive bacteria [17].

The activity on Gram-negative bacteria is much lower with minimum inhibitory concentration (MIC) values of 128 mg/mL for *E. coli*, while for a Gram-positive bacterium such as *S. aureus* it is 18 mg/mL [83].

Extracts obtained by Soxhlet extraction using ethyl acetate or ethanol show strong antifungal activity against *Trichophyton rubrum* (MIC = 0.25 mg/mL). Other extracts obtained by Soxhlet extraction or supercritical fluids report moderate activity (MIC = 0.50 mg/mL) against *Microsporum canis*, *Microsporum gypseum*, or *Trichophyton rubum*, and low effectiveness (MIC = 1.00 mg/mL) against *Epidermophyton floccosum* (50 mg/mL) and *Microsporum canis*, *Microsporum gypseum*, or *Trichophyton rubrum*, and low effectiveness (MIC = 1.00 mg/mL) against *Epidermophyton floccosum*, *M. canis*, *M. gypseum*, *Trichophyton mentagrophytes*, or *Trichophyton rubrum* [31]. Hydroethanolic or methanolic extracts report the ability for guayusa to be used against *Microsporum canis* or *Candida albicans* [84].

There is research focused on guayusa’s effectiveness in the control of pathogens associated with periodontal conditions, which validates the ancestral use of the infusion in oral cleaning [2,7,40]. The assays showed antimicrobial activity against *Porphyromonas gingivalis* ATCC 33277 and *Prevotella intermedia* ATCC 25611, with a minimum inhibitory concentration (MIC) of 1 mg/mL [27].

#### 2.7.6. Fertility Increase

Guayusa infusion has been used to treat infertility since the earliest records of its use [2]. Its efficacy has been evaluated in ethanolic extracts by oral administration in rats, the results of which reflect increased ovarian and uterine weight and increased serum estradiol levels at low doses, from 9 mg/kg [2,85].

The specific compounds that cause the results in fertility have not been determined. The reported effects are attributable to the structural similarity of phytoestrogens with 17-β estradiol, which makes them able to bind to the receptor of this molecule, with direct effects on the production of luteinizing hormones and follicle stimulating hormones [85]. Finally, there is the content of flavonoids, which are metabolites that possess hypoazotemic, hypotensive, estrogenic, and spasmolytic properties [55].

These results reveal a promising use for the treatment of anovulatory infertility, as well as great potential as hormone replacement therapy in menopause [85].

#### 2.7.7. Toxicity of Guayusa

Kapp et al., in 2016, in a study conducted on rats determined that the median lethal dose (LD_50_) was 5000 mg/kg, considering guayusa as a low toxicity plant. In this same study at high doses of 1200 mg/kg and 2500 mg/kg per day, over a period of 90 days, effects such as weight loss were determined, with no significant internal damage observed [55].

A study carried out by Paladines et al. in the year 2021 on an energizing drink of guayusa showed low toxicity, without showing toxicity at doses of 2000 mg/kg [86].

No toxicity studies have been carried out on pregnant women or young children.

## 3. Discussion

Both the traditional consumption and the industrial use of guayusa have exploited the stimulant properties of the plant as the main benefit. In this review, it has been established that other biological activities such as antioxidant, anti-aging, anti-diabetes, and antimicrobial properties are attributable to guayusa, which, when studied in greater depth would give us the possibility of diversifying its industrial use.

As far as secondary metabolites are concerned, the latest research has shown a high diversity beyond caffeine. Secondary metabolites such as polyphenols, triterpenes, tetraterpenes, and other alkaloids present in guayusa are already used in various medicinal therapies and are of interest in several applications.

The plant grows almost exclusively in Ecuador, so it is in this country where there are crops and industrial processes. The sales of guayusa currently reaches ten million USD with growth forecast; however, its exploitation has been focused exclusively on the preparation of energy drinks, leaving aside other applications that are addressed in this review, leaving open the possibility of further increasing the industrial growth forecasts.

Finally, given that this plant was domesticated more than a thousand years ago by the indigenous peoples of the Ecuadorian Amazon, current exploitation has not caused any survival problems for the species; however, an analysis is needed regarding intensive cultivation, since at the moment the farming systems have been able to produce enough for the species to survive.

## 4. Materials and Methods

For this bibliographic compilation, a search, which was non-exclusive of language or quartile of impact, had an open publication time range, and considered the information available up to the date of writing due to the scarce records of research on guayusa, was performed. “*Ilex guayusa*” was used as a search key along with the terms “bioactivity”, “antioxidant”, “composition”, “activity”, “traditional uses”, “compounds”, and “ethnobotany” combined or with slight variations. Complementary publications were included, with topics such as the particularities of the region, bioactive compounds, and cultural aspects, among others.

The search was carried out using search engines integrated with open access and subscription databases or repositories, such as MDPI, Science Direct, Wiley Online Library, Web of Science, Taylor and Francis Online, Springer Nature Link, Scopus, PubMed, and PubMed Central. All articles were collected in the period from June to December 2024.

Much of the original information concerning botanical particularities, reproductive viability, or distribution in the wild belongs to old research, due to the lack of recent publications.

## 5. Conclusions

Due to the abundant presence of bioactive molecules, of which we highlight three groups of importance, alkaloids, terpenes, and phenolic compounds, guayusa exhibits a varied biological activity, including: stimulant, antioxidant, anti-diabetic, antimicrobial, and cosmetic, among others. This makes it possible for it to be projected as one of the bioproducts with the greatest commercialization potential in industries such as functional food, pharmaceuticals, and cosmetics.

To these benefits we must add that it is a species whose distribution is almost exclusive to Ecuador, and that it is a species that has been domesticated and cultivated for millennia by ancestral peoples and, therefore, its industrialization would not represent a danger to its survival and would serve as a source of income for communities in the upper Amazon region of Ecuador.

## Data Availability

Not applicable.
